# Relationship between coffee consumption and stroke risk in Korean population: the Health Examinees (HEXA) Study

**DOI:** 10.1186/s12937-017-0232-y

**Published:** 2017-01-31

**Authors:** Jeeyoo Lee, Ji-Eun Lee, Yuri Kim

**Affiliations:** 1 Department of Nutritional Science and Food Management, Ewha Womans University, 52 Ewhayeodae-gil, 03760 Seodaemun-gu, Seoul South Korea; 2 Department of Physical Education, College of Education, Chung-Ang University, 06974 Seoul, Republic of Korea

**Keywords:** stroke, coffee, Korean, HEXA

## Abstract

**Background:**

Although coffee consumption is increasing rapidly, the results of previous studies regarding the association between coffee consumption and stroke risk have been conflicting. This was a multi-center cross-sectional study that aimed to evaluate the relationship between coffee consumption and stroke risk in Korean population.

**Methods:**

Data were obtained from the Health Examinees (HEXA) Study, which involved 146,830 individuals aged 40–69 years. Coffee consumption was categorized as none, < 1 cup/day, 1 to < 3 cups/day, and ≥ 3 cups/day. We used logistic regression models to examine the association between coffee consumption and the risk of stroke while controlling for potential confounders and performed subgroup analyses.

**Results:**

After adjusting for age and various possible confounders, high coffee consumption was associated with a 38% lower odds ratio for stroke in women (none vs. ≥ 3 cups/day: OR, 0.62; 95% CI 0.47-0.81; *P* for trend < 0.0001). No significant association was found in men (none vs. ≥ 3 cups/day: OR, 0.84; 95% CI, 0.66-1.07; *P* for trend = 0.1515). In analyses stratified by covariates, an inverse association between coffee consumption and stroke risk was most evident among healthy women who were younger, non-obese, non-hypertensive, non-diabetic, non-smokers, and non-alcohol drinkers.

**Conclusion:**

Our results suggest that higher coffee consumption may have protective benefits with regards to stroke risk in middle-aged Korean women.

## Background

Coffee is one of the most popular beverages consumed worldwide [1]. According to a report issued by the Ministry of Agriculture, Food and Rural Affairs, Korea’s per capita coffee consumption was 3.38 kg in 2011, representing a 17% increase from 2.91 kg in 2008; this rate of increase is greater than that in most other countries [2]. The Korea Health Statistics 2014 report, published by the Ministry of Health and Welfare and the Korea Centers for Disease Control and Prevention, estimates Korean per capita coffee consumption at 11.99 cups/week [3]. As coffee has become more popular, the importance of coffee consumption to public health has received increasing attention.

Caffeine and polyphenol, the rich bioactive compounds in coffee, may play important roles in reducing the risk of stroke through several complex mechanisms, including increased antioxidant activity, insulin sensitivity, hypocholesterolemia, and vascular endothelial function [4, 5]. Several epidemiological studies have suggested that high coffee consumption has protective effects on the risks of Parkinson’s disease, Alzheimer’s disease, cardiovascular disease, and diabetes mellitus [6, 7]. In 2014, cerebrovascular disease was the third leading cause of death in Korea, and the general public became more aware of the dangers of stroke, including its high mortality rate and serious side effects [8].

Data on the association between high coffee consumption and the risk of stroke has been inconsistent [9–11]. A meta-analysis of 11 cohort studies conducted in Japan (n = 2), the U.S (n = 7) and Europe (n = 7) yielded a U-shaped curve, which showed that low and high coffee consumption are both associated with an increased risk of stroke [12]. This distribution may be due to differences in study populations and designs. The type and pattern of coffee intake in Korea may differ considerably from those in other countries. For instance, filtered or brewed coffee is the most popular form of coffee consumed in the U.S and Europe [7],whereas Koreans tend to consume more soluble types of coffees (usually an instant mix containing non-dairy creamer and sugar) rather than brewed coffee [13]. Thus, results from studies done in other countries may not be fully applicable to the Koreans population. In addition, no large population-based study has investigated the association between coffee consumption and risk of stroke in Korea. The aim of this study was to examine the relationship between coffee consumption and the risk of stroke in a large Korean population using data from the Health Examinees (HEXA) baseline Study. The results may be useful in developing daily coffee consumption recommendations for stroke prevention.

## Methods

### Study population

We used data from the HEXA study, a large-scale community-based prospective cohort study involving 173,357 subjects that is part of a larger Korean Genome and Epidemiology Study (KoGES). Only base line information from the HEXA study was used in the present study. Briefly, the study was conducted in Korea from 2004 through 2013 by the K-CDC with the main objective of identifying general characteristics of public health with regard to major chronic diseases. According to standardized study criteria, subjects were recruited from 35 major hospitals and local health examination centers. All of the participants received a detailed description of the study beforehand and voluntarily signed an agreement form. The enrolled subjects included men and women who ranged in age from 40 to 69 years and who participated in a clinical health check-up program. Eligible subjects were required to reply to a structured questionnaire, which was consistently conducted in a consistent manner by trained staff. The questionnaire provided information regarding the general characteristics, medical history, medication usage, family history, lifestyle factors, diet (food frequency questionnaire), and physical activity of each subject. For women, the questionnaire also included a section regarding reproductive factors. Detailed information about the HEXA Study has been reported elsewhere [14].

At baseline, 173,357 subjects were included in the HEXA Study. We excluded individuals who did not respond about stroke diagnosis (n = 667), those with a family history of stroke (n = 23,715), and individuals for whom information on habitual coffee consumption was missing (n = 2,145). After these exclusions, 146,830 individuals (50,439 men and 96,391 women) were included in the final analysis. All participants provided written informed consent at the time they took the baseline HEXA survey. The protocol of the current study was approved by the Institutional Review Board of the Ewha Womans University, in Seoul, Korea (IRB no. 109–2).

### Baseline data collection

Trained interviewers conducted the survey using a strictly structured questionnaire [15]. Data on participants’ age, education level, marital status, household income, body mass index (BMI), smoking status, alcohol consumption habits, exercise habits, and history of chronic diseases, including hypertension, diabetes mellitus, and hyperlipidemia, were collected. BMI was categorized according to the guidelines of the Steering Committee of the Regional Office for the Western Pacific Region of the World Health Organization [16]. Obesity was defined as a BMI ≥ 25.0 kg/m^2^. Diabetes was defined as a fasting blood glucose level ≥ 126 mg/dL or according to diagnosis by a doctor. Hypertension was defined as a systolic blood pressure ≥ 140 mmHg or a diastolic blood pressure ≥ 90 mmHg [17], or according to diagnosis by a doctor. Stroke was evaluated by a questionnaire question that asked participants whether they had ever been diagnosed with a stroke by a doctor. Stroke subtype (e.g., ischemic, hemorrhagic) was not specified in the questionnaire.

### Assessment of coffee consumption

Subjects’ coffee consumption was estimated using data from a 106-item semi-quantitative food frequency questionnaire developed for the Korean population. The reliability and validity of this questionnaire were established in a prior Korean population-based study [15]. Participants were asked to indicate how many cups of coffee they drank per day, per week, or per month, and the average volume per drink (1/2 cup, 1 cup, 2 cups), during the last year. The questionnaire did not solicit information on the type of coffee consumed (e.g. instant, drip, or filtered) or caffeine content. Subjects were grouped subjects based on their average coffee consumption as follows: none, < 1 cup a day, 1 to < 3 cups/ day, and ≥ 3 cups a day.

### Statistical analysis

All analyses were stratified by sex, and SAS software (version 9.4; SAS Institute Inc., Cary, NC, USA) was used for the main analyses. The cutoff for statistical significance was set at *P* < 0.05. The chi-squared test was used to compare subjects’ general characteristics according to frequency of coffee consumption. We used regression models to assess the association between coffee consumption and stroke risk. Binary logistic regression was used to calculate the odds ratios (OR) and the associated 95% confidence intervals (CI) for each coffee consumption category, and tests to assess trends were performed. The analyses were adjusted for the following potential confounders, based on the literature [10, 12, 18–20] and participants’ demographic and clinical characteristics: age, education level, alcohol consumption, regular exercise, BMI, smoking, caloric consumption, and diagnosed hypertension, diabetes, and hyperlipidemia (Table 1). In addition, stratified analyses according to alcohol consumption, hypertension, BMI, smoking status, diabetes, and age were performed to further investigate the interactions between coffee consumption and these variables in the association with stroke.

**Table 1:** Baseline characteristics of the Health Examinees (HEXA) Study subjects by coffee consumption

										N (%)
Daily Coffee Consumption, No. of Cups										
	Male (*n =* 50439)					Female (*n =* 96391)				
variable	None	<1	1 to 3	≥3	*P*-value^†^	None	<1	1 to 3	≥3	*P*-value
Number	6493	10141	19473	14332		18012	23015	40246	15118	
Age group (years)					<.0001					<.0001
49	1844 (28.4)	3277 (32.3)	6349 (32.6)	6090 (42.5)		4536 (25.2)	8396 (36.5)	16276 (40.4)	8097 (53.6)	
50-59	2240 (34.5)	3619 (35.7)	6876 (35.3)	5037 (35.2)		7347 (40.8)	9305 (40.4)	15865 (39.4)	5415 (35.8)	
60-69	2127 (32.8)	2887 (28.5)	5605 (28.8)	2948 (20.6)		5574 (31.0)	4929 (21.4)	7558 (18.8)	1530 (10.1)	
70+	282 (4.3)	358 (3.5)	643 (3.3)	257 (1.8)		555 (3.1)	385 (1.7)	547 (1.4)	76 (0.5)	
Marital status					<.0001					<.0001
Married	5693 (87.7)	8742 (86.2)	17540 (90.1)	12828 (89.5)		14231 (79.0)	18193 (79.1)	33261 (82.6)	12450 (82.4)	
Single	457 (7.0)	775 (7.6)	1108 (5.7)	977 (6.8)		2931 (16.3)	3245 (14.1)	5403 (13.4)	2097 (13.9)	
Education level					<.0001					<.0001
Under middle school	1733 (26.7)	2300 (22.7)	4395 (22.6)	3058 (21.3)		8372 (46.5)	8802 (38.2)	14283 (35.5)	4164 (27.5)	
High school	2433 (37.5)	3985 (39.3)	7653 (39.3)	6002 (41.9)		6258 (34.7)	9509 (41.3)	16963 (42.2)	6949 (46.0)	
College or more	2164 (33.3)	3664 (36.1)	7039 (36.2)	5005 (34.9)		2411 (13.4)	3856 (16.8)	7597 (18.9)	3638 (24.1)	
Household Income (10,000 won/month)					<.0001					<.0001
< 200	1817 (28.0)	2637 (26.0)	4725 (24.3)	3134 (21.9)		6362 (35.3)	6567 (28.5)	11286 (28.0)	3664 (24.2)	
200-400	2237 (34.5)	3628 (35.8)	7608 (39.1)	5927 (41.4)		5522 (30.7)	7631 (33.2)	14634 (36.4)	5719 (37.8)	
> 400	1398 (21.5)	2276 (22.4)	4631 (23.8)	3643 (25.4)		2481 (13.8)	4074 (17.7)	8100 (20.1)	3701 (24.5)	
Body mass index (kg/m^2^)					<.0001					<.0001
< 25	4333 (66.7)	6061 (59.8)	11528 (59.2)	8370 (58.4)		13385 (74.3)	16223 (70.5)	27883 (69.3)	10560 (69.9)	
≥ 25	2133 (32.9)	4021 (39.7)	7879 (40.5)	5925 (41.3)		4568 (25.4)	6681 (29.0)	12252 (30.4)	4505 (29.8)	
Smoking status					<.0001					<.0001
Non-smoker	2694 (41.5)	3500 (34.5)	5439 (27.9)	2547 (17.8)		17596 (97.7)	22285 (96.8)	38765 (96.3)	13875 (91.8)	
Ex-smoker	2651 (40.8)	4204 (41.5)	8234 (42.3)	4935 (34.4)		152 (0.8)	282 (1.2)	492 (1.2)	304 (2.0)	
Current smoker	1130 (17.4)	2420 (23.9)	5766 (29.6)	6830 (47.7)		205 (1.1)	322 (1.4)	848 (2.1)	895 (5.9)	
Alcohol consumption					<.0001					<.0001
Non-drinker	1725 (26.6)	1913 (18.9)	3683 (18.9)	2976 (20.8)		14679 (81.5)	15739 (68.4)	25434 (63.2)	8452 (55.9)	
Ex-drinker	704 (10.8)	806 (8.0)	1269 (6.5)	902 (6.3)		361 (2.0)	563 (2.5)	748 (1.9)	348 (2.3)	
Current drinker	4051 (62.4)	7407 (73.0)	14491 (74.4)	10438 (72.8)		2908 (16.1)	6604 (28.7)	13953 (34.7)	6276 (41.5)	
Regular exercise					<.0001					<.0001
No	2620 (40.4)	4065 (40.1)	8332 (42.8)	7068 (49.3)		8816 (49.0)	10531 (45.8)	20246 (50.3)	8309 (55.0)	
Yes	3858 (59.4)	6054 (59.7)	11095 (57.0)	7249 (50.6)		9143 (50.8)	12410 (53.9)	19910 (49.5)	6787 (44.9)	
Hypertension					<.0001					<.0001
No	4174 (64.3)	6400 (63.1)	12382 (63.6)	9967 (69.5)		12613 (70.0)	16923 (73.5)	30334 (75.4)	12243 (81.0)	
Yes	2318 (35.7)	3737 (36.9)	7089 (36.4)	4363 (30.4)		5383 (29.9)	6078 (26.4)	9897 (24.6)	2865 (19.0)	
Diabetes mellitus					<.0001					<.0001
No	5352 (82.4)	8487 (83.7)	16481 (84.6)	12496 (87.2)		15903 (88.3)	20728 (90.1)	36870 (91.6)	14089 (93.2)	
Yes	1140 (17.6)	1650 (16.3)	2987 (15.3)	1835 (12.8)		2101 (11.7)	2281 (9.9)	3362 (8.4)	1024 (6.8)	
Hyperlipidemia					0.0007					<.0001
No	5498 (84.7)	8451 (83.3)	16240 (83.4)	11865 (83.8)		14356 (79.7)	18489 (80.3)	32401 (80.5)	12553 (83.0)	
Yes	989 (15.2)	1687 (16.6)	3216 (16.5)	2465 (17.2)		3648 (20.3)	4514 (19.6)	7831 (19.5)	2561 (16.9)	

^†^ *p*-values were calculated by chi-square test

## Results

Table 1 describes the participants’ basic demographic characteristics according to sex and daily coffee consumption (none, n = 24,505; < 1 cup/day, n = 33,156; 1 to < 3 cups/day, n = 59,719; ≥ 3 cups/day, n = 29,450). The prevalence of stroke in the study population as a whole was 1.3%, the prevalence for men was 1.54%, and that for women was 1.14% (data not shown). In men and women, several variables (age group, marital status, educational level, household income, BMI, alcohol consumption, smoking status, regular exercise, hypertension, diabetes, and hyperlipidemia) differed significantly according to coffee consumption. Men and women with higher frequencies of coffee consumption tended to be younger and to have more education, higher incomes, and higher BMIs. They were also somewhat more likely to be current smokers and current drinkers and to perform less regular exercise (*P* for all < 0.0001). Subjects with higher frequencies of coffee consumption tended to have a lower prevalence of hypertension, diabetes mellitus, and hyperlipidemia.

The ORs and 95% CIs for stroke prevalence according to coffee consumption are presented in Table 2. Among men, no significant association was found between coffee consumption and the risk of stroke. However, among women, inverse associations were found between all coffee consumption groups and stroke prevalence. Although further adjustment attenuated this association, the odds ratio for stroke was 38% lower among women who consumed ≥ 3 cups of coffee per day than among non-coffee drinkers (OR, 0.62; 95% CI, 0.47-0.81).

**Table 2:** Odd ratios and 95% confidence intervals of stroke by coffee consumption in the Health Examinees (HEXA) Study

Daily Coffee Consumption, No. of Cups					
	None	<1	1 to 3	≥3	*P* for trend
Male (*N =* 50439)	(*N =* 6493)	(*N =* 10141)	(*N =* 19473)	(*N =* 14332)	
Model 1	1.00	0.83 (0.66-1.04)	0.78 (0.63-0.96)	0.58 (0.46-0.73)	<0.0001
Model 2	1.00	0.95 (0.75-1.21)	0.93 (0.75-1.15)	0.82 (0.64-1.04)	0.1049
Model 3	1.00	0.95 (0.75-1.20)	0.92 (0.75-1.14)	0.84 (0.66-1.07)	0.1515
Female (*N =* 96391)	(*N =* 18012)	(*N =* 23015)	(*N =* 40246)	(*N =* 15118)	
Model 1	1.00	0.54 (0.45-0.66)	0.44 (0.37-0.52)	0.34 (0.26-0.44)	<0.0001
Model 2	1.00	0.67 (0.56-0.82)	0.59 (0.50-0.71)	0.59 (0.45-0.78)	<0.0001
Model 3	1.00	0.68 (0.56-0.82)	0.60 (0.50-0.72)	0.62 (0.47-0.81)	<0.0001

Model 1: Unadjusted

Model 2: adjusted for age, education, alcohol consumption, regular exercise, BMI, smoking and total energy

Model 3: adjusted for age, education, alcohol consumption, regular exercise, BMI, smoking, total energy, hypertension, diabetes mellitus and hyperlipidemia

The results of covariate-stratified analyses of the association between stroke prevalence and coffee consumption are presented in Table 3. Among men, no significant association was found between coffee consumption and stratified covariates. Among women, significant associations were found according to the strata of age, BMI, hypertension, diabetes, smoking, and alcohol consumption. The inverse association of coffee consumption with stroke prevalence was most evident among healthy individuals who were younger (OR, 0.56; 95% CI, 0.42 – 0.76), non-obese (OR, 0.53; 95% CI, 0.36 – 0.77), non-hypertensive (OR, 0.52; 95% CI, 0.34 – 0.80), non-smokers (OR, 0.58; 95% CI, 0.43 – 0.77), and non-drinkers (OR, 0.61; 95% CI, 0.44 – 0.84).

**Table 3:** Odd ratios and 95% confidence intervals of stroke by coffee consumption stratified by age, body mass index (BMI), hypertension, diabetes mellitus, smoking status, and alcohol consumption in the Health Examinees (HEXA) Study

Categories of coffee consumption (cup/day)						
	no. of events	None	<1	1 to 3	≥3	*P* for trend
Male (*n =* 50439)						
^a^Age (years)						
65-	512	1.00	0.80 (0.60-1.07)	0.83 (0.64-1.07)	0.75 (0.56-0.99)	0.0802
65+	252	1.00	1.25 (0.83-1.90)	1.16 (0.80-1.70)	0.93 (0.59-1.47)	0.7079
^b^BMI (kg/m^2^)						
< 25	425	1.00	1.01 (0.74-1.36)	0.88 (0.67-1.17)	0.87 (0.63-1.19)	0.2420
≥ 25	335	1.00	0.86 (0.59-1.25)	0.96 (0.69-1.34)	0.80 (0.55-1.17)	0.4197
^c^Hypertension						
Nonhypertensive	368	1.00	1.01 (0.69-1.48)	0.86 (0.60-1.22)	0.86 (0.59-1.26)	0.2839
Hypertensive	396	1.00	0.90 (0.67-1.22)	0.96 (0.73-1.25)	0.81 (0.59-1.12)	0.3156
^d^Diabetes mellitus						
Nondiabetic	573	1.00	1.02 (0.78-1.34)	0.86 (0.67-1.11)	0.86 (0.65-1.13)	0.1249
Diabetic	191	1.00	0.76 (0.47-1.24)	1.11 (0.74-1.67)	0.77 (0.47-1.27)	0.8031
^e^Smoking status						
Never and past	604	1.00	1.01 (0.78-1.30)	0.96 (0.76-1.21)	0.89 (0.67-1.17)	0.3436
Current	157	1.00	0.71 (0.38-1.32)	0.76 (0.44-1.30)	0.73 (0.42-1.27)	0.4607
^f^Alcohol consumption						
Never and past	356	1.00	1.13 (0.81-1.58)	1.00 (0.74-1.36)	0.72 (0.50-1.03)	0.0636
Current	407	1.00	0.78 (0.56-1.09)	0.78 (0.58-1.05)	0.85 (0.61-1.18)	0.4576
Female (*n =* 96391)						
^a^Age (years)						
65-	552	1.00	0.66 (0.53-0.84)	0.58 (0.47-0.72)	0.56 (0.42-0.76)	<.0001
65+	211	1.00	0.64 (0.45-0.62)	0.59 (0.42-0.83)	0.59 (0.30-1.17)	0.0022
^b^BMI (kg/m^2^)						
< 25	437	1.00	0.68 (0.53-0.88)	0.59 (0.46-0.74)	0.53 (0.36-0.77)	<.0001
≥ 25	324	1.00	0.69 (0.51-0.94)	0.64 (0.48-0.85)	0.74 (0.50-1.10)	0.0203
^c^Hypertension						
Nonhypertensive	364	1.00	0.54 (0.39-0.75)	0.60 (0.45-0.80)	0.52 (0.34-0.80)	0.0007
Hypertensive	399	1.00	0.77 (0.60-0.98)	0.60 (0.48-0.76)	0.70 (0.49-0.99)	0.0001
^d^Diabetes mellitus						
Nondiabetic	613	1.00	0.64 (0.51-0.80)	0.57 (0.47-0.70)	0.66 (0.49-0.88)	<.0001
Diabetic	150	1.00	0.80 (0.52-1.22)	0.74 (0.50-1.11)	0.29 (0.11-0.74)	0.0132
^e^Smoking status						
Never and past	736	1.00	0.67 (0.55-0.82)	0.59 (0.49-0.71)	0.58 (0.43-0.77)	<.0001
Current	23	1.00	2.09 (0.30-14.78)	2.88 (0.54-15.25)	3.30 (0.61-17.91)	0.1571
^f^Alcohol consumption						
Never and past	612	1.00	0.66 (0.53-0.82)	0.61 (0.50-0.74)	0.61 (0.44-0.84)	<.0001
Current	147	1.00	0.78 (0.47-1.29)	0.59 (0.37-0.95)	0.63 (0.35-1.13)	0.0417

^a^Adjusted by education, alcohol consumption, regular exercise, BMI, smoking, total energy, hypertension, diabetes mellitus, and hyperlipidemia

^b^Adjusted by age, education, alcohol consumption, regular exercise, smoking, total energy, hypertension, diabetes mellitus, and hyperlipidemia

^c^Adjusted by age, education, alcohol consumption, regular exercise, BMI, smoking, total energy, diabetes mellitus, and hyperlipidemia

^d^Adjusted by age, education, alcohol consumption, regular exercise, BMI, smoking, total energy, hypertension, and hyperlipidemia

^e^Adjusted by age, education, alcohol consumption, regular exercise, BMI, total energy, hypertension, diabetes mellitus, and hyperlipidemia

^f^Adjusted by age, education, regular exercise, BMI, smoking, total energy, hypertension, diabetes mellitus, and hyperlipidemia

## Discussion

In the present study, higher coffee consumption was found to be inversely associated with the prevalence of stroke in women. No dose–response relationship was found between coffee consumption and the prevalence of stroke. After adjustment for other risk factors, high coffee consumption was associated with a significantly lower prevalence of stroke in women.

According to the American Heart Association (AHA) and the American Stroke Association (ASA) in 2015, risk factors for stroke include age, heredity, race, sex, transient ischemic attacks (TIAs), high blood pressure, diabetes mellitus, high blood cholesterol, other heart diseases, physical inactivity and obesity, and poor diet [21, 22]. Green tea, oolong tea, and black teas are the most consumed teas in Asia and several Western countries and have been reported to be associated with a lower risk of cardiovascular disease and stroke [23–25]. Coffee contains a complex mixture of bioactive substances, and reported associations of coffee consumption with cardiovascular diseases and diabetes have been inconsistent [12, 26, 27].

A meta-analysis of epidemiological studies showed that coffee consumption (≥4 cups/day) was inversely associated with the risk of stroke [28]. Another meta-analysis [12] found that the association between the two was U-shaped. In cohort studies of women in the US [10] and Sweden [9], moderate consumption of coffee reduced the risk of stroke by approximately 20%. In a cohort study conducted in the Japan, subjects aged ≥ 45 years who consumed coffee (≥4 cups per day) showed a reduced stroke risk compared with non-coffee drinkers. An American cross-sectional study suggested that heavier daily coffee consumption had a beneficial effect on stroke prevalence [29], whereas other studies have shown either no association or an increase in stroke risk according to high or low coffee consumption [30–32]. The variance in these findings may be partly due to differences in the amount of caffeine that was consumed and the different methods of coffee preparation. It is also important to note that these findings must be interpreted relative to the cultural aspects of coffee consumption, which can vary greatly.

The findings from a cohort study of Swedish women and the Nurses’ Health Study are consistent with our results [9, 10]. In addition, the Health Professionals Follow-up Study showed no significant association between coffee consumption and stroke in men. However, a Japanese cohort study found a significant inverse relationship between coffee consumption and stroke risk in men who drank 3 cups of coffee per day [25]. Although the underlying mechanism for the difference in the results between sexes is unknown, Nagata et al. [33] and Ferrini et al. [34] reported that caffeine intake may be beneficially associated beneficially with plasma estrogen, plasma estradiol, and sex hormone-binding globulin levels, as it is inversely related to testosterone level in postmenopausal women. Physical, physiological, hormonal, and lifestyle differences between the sexes may influence the relationship between coffee intake and stroke risk. Surprisingly, the odds ratio for stroke risk was 0.29 (95% CI, 0.11 - 0.74) among diabetic women who consumed ≥ 3 cups of coffee per day as compared to non-coffee drinkers. That is lower than the odds ratio among non-diabetic women (OR, 0,66; 95% CI, 0.49 – 0.88).

The anti-diabetic and anti-stroke effects of coffee may be explained by the bioactive compounds or nutrients contained in coffee. Coffee contains chlorogenic acids, which may contribute to its anti-diabetic effect by reducing cholesterol levels, blood glucose levels, and insulin resistance [35]. In addition, caffeine, a major ingredient in coffee, increases peripheral vascular resistance and blood pressure [36], but a large prospective study revealed an inverse U-shaped association between coffee consumption and the incidence of hypertension [37]. In a meta-analysis of randomized controlled trials, chronic coffee intake was associated beneficially with hypertension, an important main risk factor for stroke [38]. Other phenolic compounds in coffee, including chlorogenic acid, ferulic acid, quinides, and *p*-coumaric acid, possess high antioxidant abilities, which may help reduce not only endothelial dysfunction [39, 40], but also low-density lipoprotein oxidation [41]. Furthermore, the magnesium, potassium, trigonelline, and niacin in coffee have been associated with enhanced insulin sensitivity, which may prevent the progression of diabetes mellitus [42, 43]. More precise anti-diabetic mechanisms of coffee require further investigation

The present study has several limitations. First, because it was cross-sectional in nature, the cause and effect relationship between coffee consumption and the stroke risk could not be investigated. These results may not reflect long-term coffee consumption patterns, as the study was cross-sectional. To increase the methodological strength of the analyses, adjustments for several known confounding factors, sex stratification, and subgroup analyses were performed. Second, information about stroke types (ischemic, hemorrhage) was not collected, and it is possible that different stroke types may be affected differently by coffee consumption. Third, different types of coffee (caffeinated vs. decaffeinated) and preparation (e.g., filtered, boiled, espresso, and instant), as well as the addition of sugar, milk, and/or powdered creamer, were not considered in the analyses. This heterogeneity may partly explain the differences between studies. Lastly, because the original study did not account for these variables, we could not consider other stroke risk factors deemed important by the AHA and the ASA.

The major strength of this study is its inclusion of a large number of subjects and stroke cases, which provided high statistical power for the analysis of the association between coffee consumption and stroke prevalence. The present study is the first to explore the association between coffee consumption and the prevalence of stroke in a Korean population. Although it was cross-sectional, these results have value as the coffee consumption patterns of Koreans particularly intake level and preparation method – differ from those of other populations. Koreans do not drink decaffeinated coffee, and they often drink instant coffee with sugar and powdered creamer. A previous study suggested that consumption of coffee prepared in this manner is associated with an increased risk of metabolic syndrome risk in Koreans [44]. Although the coffee consumption data in the present study included coffee prepared in this manner, coffee intake was inversely associated with the risk of stroke. Further research is needed to investigate whether this association is attributable to coffee alone. Some evidence suggests that the rate of caffeine metabolism differs between races [45, 46].

## Conclusion

Results from this cross-sectional study imply that high coffee consumption, as currently practiced by Koreans, may confer a protective relationship with regards to the development of stroke in this population. This association should be investigated further in validated long-term cohort studies with large numbers of subjects from different ethnic groups.

## Abbreviations

AHA: American heart association
ASA: American stroke association
BMI: Body mass index
HEXA: The health examinees
K-CDC: Korean centers for disease control and prevention
TIAs: Transient ischemic attacks
